# Job preferences among clinical officers in public sector facilities in rural Kenya: a discrete choice experiment

**DOI:** 10.1186/s12960-015-0097-0

**Published:** 2016-01-08

**Authors:** Toshio Takemura, Karina Kielmann, Duane Blaauw

**Affiliations:** Institute for Global Health and Development, Queen Margaret University, Edinburgh, EH21 6UU UK; Centre for Health Policy/MRC Health Policy Research Group, School of Public Health, Faculty of Health Sciences, University of the Witwatersrand, Johannesburg, South Africa

**Keywords:** Mid-level workers, Clinical officers, Job preferences, Retention, Discrete choice experiment, Kenya

## Abstract

**Background:**

Clinical officers (COs), a mid-level cadre of health worker, are the backbone of healthcare provision in rural Kenya. However, the vacancy rate for COs in rural primary healthcare facilities is high. Little is known about factors motivating COs’ preferences for rural postings.

**Methods:**

A discrete choice experiment (DCE) questionnaire was used with 57 COs at public health facilities in nine districts of Nyanza Province, Kenya. The questionnaire was developed on the basis of formative qualitative interviews with COs (*n* = 5) and examined how five selected job attributes influenced COs’ preferences for working in rural areas. Conditional logit models were employed to examine the relative importance of different job attributes.

**Results:**

Analysis of the qualitative data revealed five important job attributes influencing COs’ preferences: quality of the facility, educational opportunities, housing, monthly salary and promotion. Analysis of the DCE indicated that a 1-year guaranteed study leave after 3 years of service would have the greatest impact on retention, followed by good quality health facility infrastructure and equipment and a 30% salary increase. Sub-group analysis shows that younger COs demonstrated a significantly stronger preference for study leave than older COs. Female COs placed significantly higher value on promotion than male COs.

**Conclusions:**

Although both financial incentives and non-financial incentives were effective in motivating COs to stay in post, the study leave intervention was shown to have the strongest impact on COs’ retention in our study. Further research is required to examine appropriate interventions at each career stage that might boost COs’ professional identity and status but without leading to larger deficits in the availability of generalist COs.

## Background

The World Health Organization (WHO) recommends the utilization of mid-level workers (MLWs) to increase access to health workers in rural areas [1]. Since their training period is relatively short, and their remuneration is lower than that of physicians, MLWs are seen to be more financially advantageous in resource-limited settings [2]. In fact, 25 of 47 countries in sub-Saharan African countries have introduced MLWs who take on many of the functions of medical doctors especially in poorly served regions [2]. In Kenya, for example, clinical officers (COs) make up 6% of the total health workforce. While they function as the backbone of healthcare provision especially in rural areas [3], many COs express dissatisfaction with their jobs due to low remuneration, poor career progress and limited educational opportunities especially in rural areas [4] which has impacted on rural retention and attraction. For example, a recent workload analysis among doctors, nurses and COs in Kenya indicates the severe shortage of COs at rural facilities [5].

Although there are published guidelines suggesting policies to address health worker attraction and retention in remote and rural areas [1], the evidence base for effective strategies and interventions to promote retention of health workers in low- and middle-income countries is relatively weak [1, 6]. Discrete choice experiments (DCEs) have been put forward as one promising methodology to explore these issues in developing countries [7]. A DCE is a quantitative method used to assess individuals’ relative valuation of attributes by requesting them to make choices from a set of different hypothetical alternatives [8]. According to a DCE guide by WHO, the World Bank and USAID [9], the tool can be used to investigate the impact of factors that influence retention of the health workforce in rural areas. The method allows researchers to estimate the strength of preferences for specific job attributes [10].

While many studies have examined factors for health worker retention in remote areas [11], most existing research has focused on physicians and nurses. Several studies on MLWs have assessed their performance as non-physician clinicians [12, 13], but there are few studies that examine the retention of MLWs [14]. This is the first study to our knowledge that uses the DCE methodology to explore job preferences of in-service COs, a critically important cadre in the health workforce of many African countries including Kenya.

Kenya is 1 of 57 countries deemed to have a human resources for health (HRH) crisis [15]: a 2010 report indicates the density of doctors, nurses and midwives to be just 1.44 per 1000 population, far below the WHO minimum requirement for service delivery [16]. The Government of Kenya (GOK) has realized that the shortage of human resources is a bottleneck to the expansion of priority health services including those addressing the burden of HIV/AIDS, TB and malaria [17].

Kenya has been facing the challenges of attraction and retention of health workers in rural areas. A verification exercise in 2004 revealed that more than half of health workers are working in urban areas while approximately 80% of the Kenyan population live in rural regions [18]. According to the GOK Human Resources for Health Strategic Plan 2009–2012, which outlines options to retain specific target cadres and develop retention packages, “…the retention problem is particularly acute in remote/hard-to-reach areas” [3, p. xiii].

COs complement doctors and support curative, preventive, promotive and rehabilitative services [19]. In 2012, there were 2167 COs posted in public sector facilities in Kenya, which is almost twice as many as physicians [20]. The Kenyan CO cadre comprises two sub-groups: general COs (registered clinical officers, RCOs) and specialist COs (SCOs; COs with further specialist training in one medical discipline) [21]. While GOK estimates that 9827 RCOs and 1229 SCOs are required by 2017, existing numbers were 1246 and 921, respectively, as of 2012 [20]. Although some COs are in charge of health centres in rural areas [3], the distribution of COs is highly skewed toward district hospitals [18]. The vacancy rate for COs in the primary healthcare level is higher than that of doctors or nurses [16], partly because attrition rates of COs in health centres are greater than in district hospitals [17]. However, little is known about COs’ job preferences for working in rural areas.

## Methods

### Study setting and respondents

**Fig. 1:**
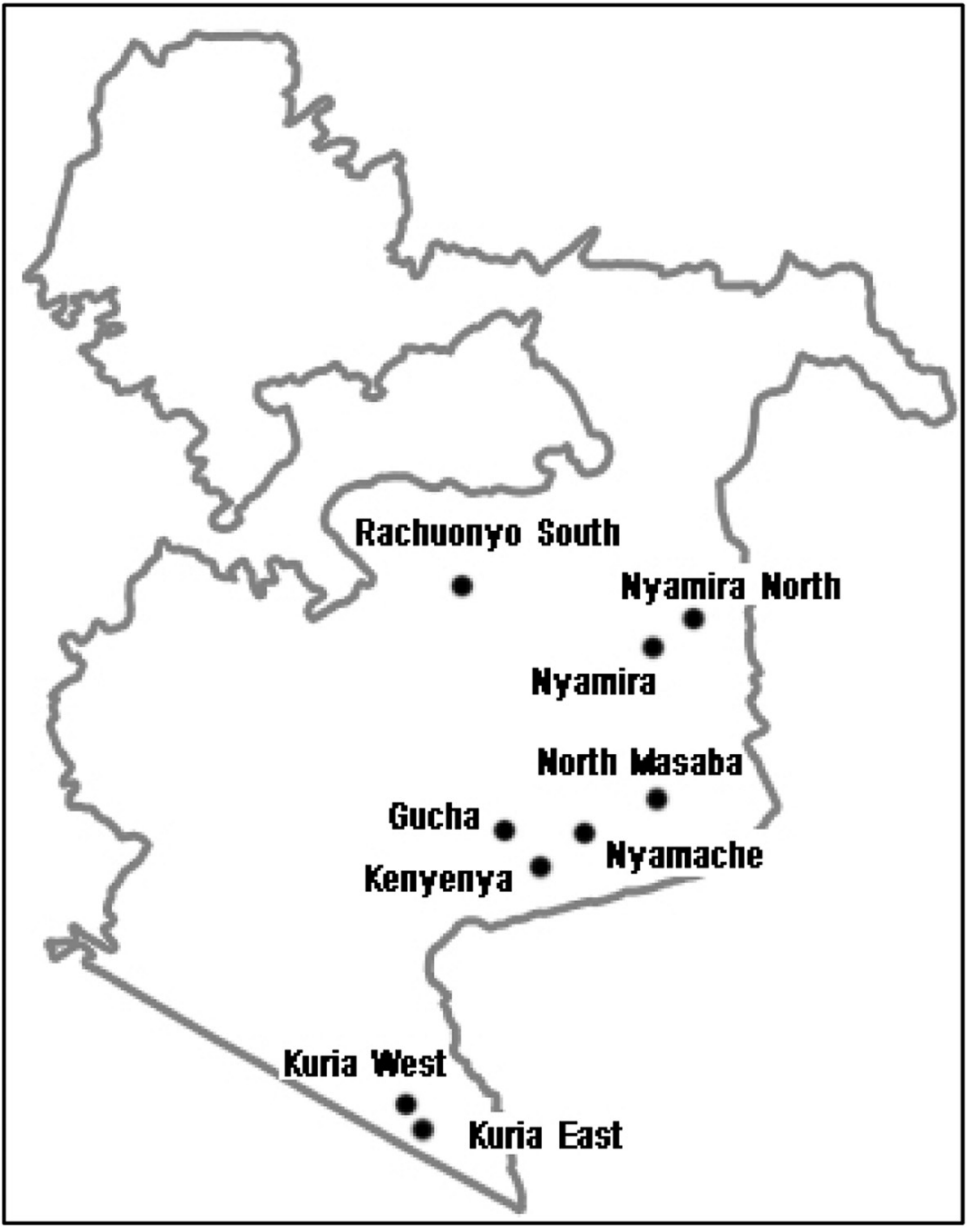
Map of Nyanza Province

This research was conducted in Nyanza Province, in the western part of Kenya (Fig. 1). The province has some of the poorest health indicators in the country with an HIV prevalence of 14.9% and an infant mortality of 95 per 1000 [22, 23]. Table 1 presents socio-demographic and health access characteristics of the selected study districts. Nyanza Province has a relatively high proportion of COs (14% of total COs in the public sector) [16].

**Table 1:** Socio-demographic characteristics and health access in the nine districts

District	Major ethnic group	Rural/urban proportion ratio^a^	No of deployed COs^b^	No of district hospitals^c^	No of sub-district hospitals^c^	No of health centres^c^
Rachuonyo South	Luo	1.9	13	1	1	2
Gucha	Kisii	16.3	9	1	0	0
Kenyenya	Kisii	16.3	5	1	0	0
Nyamache	Kisii	16.3	5	0	2	1
Nyamira	Kisii	3.3	16	1	0	14
Nyamira North	Kisii	3.3	5	0	2	8
North Masaba	Kisii	1.9	13	1	1	2
Kuria West	Kuria	7.5	12	1	1	7
Kuria East	Kuria	7.5	7	1	1	1

Source: [22, 45]

^a^As of 2009, demographic data of some districts was not segregated

^b^This information was provided directly by district officials

^c^Facilities owned by Ministry of Health are represented. Sub-district hospital is a general health facility, staffed by clinical officers and a few health practitioners [4]

### Sampling

We employed a multistage stratified cluster sampling strategy. Nine districts out of 39 were purposively selected for two reasons: (1) they were predominantly rural according to the 2009 census [22] and (2) they included various ethnic groups in Nyanza Province. Within the districts, all public health facilities that had at least one CO were selected (*n* = 31 out of 121 in Nyanza Province). All COs were subsequently invited to participate in the study. Although several studies suggest that sample size calculation for DCE depends on survey design and statistical model [24], sampling guidelines for DCE remain poorly defined [25]. Based on a previous study with a sample size of 50 pharmacy students [26], we aimed to obtain questionnaires from 50 COs. Given a 10% refusal rate or possible missing data, the required sample size for the DCE was increased to 55. We sought to survey all COs present in each facility surveyed on the day of the visit during June and July 2013.

### Instrument development

The first stage of developing a DCE tool involves identifying attributes relevant to the research question and then determining the levels of these attributes [9]. In this study, DCE attributes and levels were determined by three activities: (1) consulting other studies including those on health workers’ motivation, retention, MLWs, similar DCEs and the WHO 2010 recommendations; (2) semi-structured interviews with purposively selected COs from four districts (*n* = 5); and (3) pre-testing of the instrument with COs from one district (*n* = 3). We modified a semi-structured interview guide by Jaskiewicz and colleagues [10], which contains a range of questions about working conditions in rural regions. The aims of the interviews were twofold: (1) to obtain detailed information on COs’ job preference and (2) to identify attributes and levels for a DCE study. A total of five COs from four districts were interviewed. The interview data helped to identify the following five locally meaningful job attributes (with the related WHO [1] intervention category shown in brackets): *quality of facility* (professional and personal support), *educational opportunities* (education), *housing* (professional and personal support, financial incentives), *monthly salary* (financial incentives), and *promotion* (professional and personal support, financial incentives). Table 2 summarizes the final attributes and their levels used in this study.

**Table 2:** DCE attributes and levels for COs in Kenya, 2013

Attribute 1	Quality of facility
Level 1	Basic (e.g. unreliable electricity, equipment and drugs and supplies not always available)
Level 2	Advanced (e.g. reliable electricity, equipment and drugs and supplies always available)
Attribute 2	Education opportunity
Level 1	No guaranteed study leave
Level 2	1-year guaranteed study leave after 5 years of service
Level 3	1-year guaranteed study leave after 3 years of service
Attribute 3	Housing
Level 1	Small amount of house allowance provided, but not enough to afford basic housing^a^
Level 2	House allowance provided, enough to afford basic housing^b^
Level 3	House allowance provided, enough to afford superior housing^b^
Attribute 4	Monthly basic salary (not including allowances)
Level 1	Normal monthly basic salary^c,d ^
Level 2	Additional 10% monthly basic salary
Level 3	Additional 20% monthly basic salary
Level 4	Additional 30% monthly basic salary
Attribute 5	Promotion (number of years to be spent in facility until eligible for promotion)
Level 1	3 years
Level 2	2 years

^a^Actual amounts were not presented, assuming that current house allowance was not enough as mentioned by COs in the interviews

^b^Detailed information on basic and superior housing with participants was not provided

^c^As of 2013, entry-level COs received monthly basic salaries of KES 19 323 (USD 221) converted at a rate of (USD 1 = KES 87.31). Although monthly salary levels are different among and within COs’ job groups, there was no area difference because the monthly salary attribute in the questionnaire was described as not including allowances

^d^Following the another DCE study in Kenya [27], levels of monthly basic salary in the DCE instruments were set

The number of possible job posting scenarios depends on the number of attributes and levels. Although we used a generic design for DCE, in which the job postings were not labelled as rural or urban, respondents were requested to assume that they were looking for a new job and to choose between two advertised job postings in government health facilities in rural areas. As described in Table 2, there was one attribute with four levels, two attributes with three levels and two attributes with two levels in this study. This design generated 144 (4^1^ × 3^2^ × 2^2^) possible scenarios with different combinations of levels of the five job attributes. To reduce the number of choices, an experimental design was selected using Sawtooth Software’s Choice-Based Conjoint module (Sawtooth Software, Inc., USA). The software helps to develop DCE questionnaires that maximize level balance (inclusion of attribute levels in similar proportions) and orthogonality (minimal correlation between different attribute levels) and minimize overlap between attribute levels within one task [9, 27]. Following Jaskiewicz and colleagues [10], we used Sawtooth to generate five different versions of the questionnaire in order to improve design efficiency. Each version had 12 choice tasks with a different combination of attribute levels.

### Data collection

**Fig. 2:**
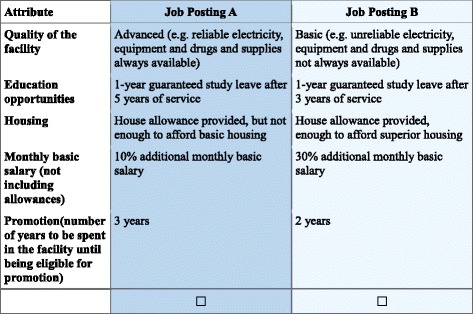
Example of choice set. Which of these two job postings do you prefer? Select one by ticking the box under the job you prefer

All respondents randomly received one version and were asked to select one of the two job scenarios from each choice task, as shown in Fig. 2. Questionnaires did not include the option of not choosing any of the alternatives. The rationale for employing a forced choice is that although an opt-out option can reduce biases in parameter estimates, it cannot provide sufficient information on respondents’ preferences for the attributes if many respondents choose the opt-out option [9].

The DCE tool included questions on respondents’ demographic characteristics and professional background. The DCE survey was conducted in English as this is the language of instruction in CO training schools. Paper-based questionnaires were administered to COs individually at their workplace during work hours. Although the researcher sought to survey all COs present in each facility on the day of the visit, some COs were not available due to their work shifts, training, maternity leave or annual leave. However, available COs were eager to participate in the survey, and no one declined. Respondents were allowed to answer at their own pace and under the researcher’s direct supervision. The survey took around 10 to 20 min to complete.

### Data analysis

The five qualitative interviews were transcribed into MS Word 2010 (Microsoft Corporation, USA). Themes emerging in relation to the overall topic of preferred job attributes were identified in a threefold manner: (1) reading the transcripts and making notes on relevant issues in the margins of the interview transcript, (2) listing out these comments in separate files and grouping them thematically and (3) selecting the most relevant themes, based on how commonly and strongly they were expressed by the respondents.

All data from the DCE questionnaires was entered and stored using Microsoft Excel 2010 (Microsoft Corporation, USA). Following this, the conditional logit (CL) model was used to analyse job preference using Stata/IC 12 module (Stata Corporation, USA). McFadden’s CL [28] is based on random utility theory where the decision maker *n* is assumed to be a rational economic individual, facing a choice among *J* alternative jobs. The individual will choose alternative job *i* over alternative job *j* if and only if *U*_*ni*_ ≥ *U*_*nj*_. The utility is not directly observable, and therefore, the model assumes that the utility function consists of two components:

$$ {U}_i = {V}_{in} + {\varepsilon}_{in} $$

The researcher can observe *V*_*in*_ while *ε*_*in*_ is unobservable and treated as a random component. Allowing *V*_*in*_ = *βx*_*ni*_, where *βx*_*ni*_ is a matrix of job attributes, the probability of choosing job *i* from *J* alternative jobs can be parametrized as the logit formula [29]:

$$ {P}_{ni} = \frac{e^{\beta {x}_{ni}}}{{\displaystyle {\sum}_j}{e}^{\beta {x}_{nj}}} $$

A CL model is obtained by assuming that the random error component is independent, identically distributed and follows the extreme value distribution [29]. Categorical variables were entered as dummy variables while salary was treated as a continuous variable. Sub-group analysis was conducted by including interaction terms between demographic variables and job attributes (salary * gender, for example). Demographic characteristics do not vary within choice sets and cannot be added into the CL regression model directly [9]. Models were run with and without the demographic interaction variables for comparison.

We also calculated willingness to pay (WTP) estimates and confidence intervals by dividing attribute coefficients by the continuous salary coefficient. WTP is useful because it can inform policymakers of the pricing of goods or services by providing information on to what extent people value them [30]. In this study, WTP refers a willingness to give up salary for an improvement in other attributes of a job.

In addition to the CL models for main effect and interactions, we further analysed the data using mixed logit models with Hole’s *mixlogit* command for Stata [31]. We ran two mixed logit (MXL) models: (i) with the salary attribute as fixed and (ii) with the salary attribute as random and lognormal. In both models, all other attributes were treated as random components following a lognormal distribution because the direction of the attribute valuation was clear from the DCE design [29]. Although some studies suggest that MXL models have advantages over standard logit models in terms of flexibility, reduction of standard error and inclusion of demographic characteristics [29], the MXL model requires a larger sample size than the CL model [25]. In this study, the results from each MXL model were not substantially different from the CL model. Given our small sample size, the main analysis applied in this study was using the CL model.

### Ethical considerations

Ethical approval was obtained from Queen Margaret University, and the study was undertaken with permission from the Provincial Director of Public Health and Sanitation Services, Nyanza Province. Written informed consent was taken from all the participants who were informed about the research and their voluntary participation.

## Results

### COs’ preferred job attributes: insights from qualitative interviews

Remuneration of Kenyan COs consists of basic salary, housing allowance, commuter allowance, health risk allowance and extraneous allowance. The basic monthly salary for a first-year CO is KES 19 323 (USD 221) as of 2013, and a hardship allowance is provided when they work in designated areas. For the five COs interviewed, critical factors relating to job preferences included salary and allowance issues but also the state of the facility, educational opportunities and possibilities for promotion.

Some COs directly mentioned the need for financial incentives in rural areas, “…so as to bring people who are working in towns to come and assist in the village.” [CO3]. Other respondents considered a *salary increase* in rural regions as compensation for a heavy workload. COs at remote facilities reported that they had to work longer due to the shortage of health workers as opposed to urban facilities which were better staffed.

Many respondents found *housing* conditions in rural and remote areas discouraging. While staff accommodation was available within some health facilities, the COs interviewed were not happy with the quality of housing. Although the GOK provides a housing allowance according to job group and location, COs were not satisfied with the amount, as expressed by a female CO based at the district hospital:

We get KES 2,300 (USD 26) […] That is too little…. As house allowance, [there should be] at least KES seven to ten [thousand] (USD 80 to 114). [CO1]

In addition to allowances, respondents suggested that the *state of the facilities* in rural areas was often lacking. They mentioned in particular the lack of equipment, shortage of drug supplies and poor infrastructure at both the district hospital and health centre level. As a result of these gaps, COs working in remote areas reported that they had to refer clients to urban hospitals even if they could manage the patients.

Health workers including COs who meet certain requirements are granted paid study leave [32]. Respondents, however, stated limited *opportunities for continuous education* in rural facilities; as one male CO in a health centre lamented:

They (government) are saying we do not have enough staff, so at the end of the day, you find yourself working throughout. You do not have time to study. [CO5]

Others pointed out the long process required to apply for study leave and the lack of tuition support for further education.

Some respondents mentioned the lack of *promotion opportunities*, especially in rural areas. One female CO based at the district hospital directly questioned the promotion system:

You are supposed to be promoted every three years, but now, you can fill the performance contracts forms yearly, but you still don’t get promoted even seven to ten years [CO1]

### Relative importance of job attributes: results from the DCE

Out of 85 public COs deployed in the study districts, 57 (67%) COs participated in the survey. The figure corresponds to 16% of COs (*n* = 356) in Nyanza Province. Details are shown in Table 3. Descriptive statistics on the demographic and professional background of COs are provided in Table 4. Sixty percent of COs surveyed was male, which was almost the same as the national level [20]. The mean age of COs was 33.0 years with a standard deviation of 7.6 years, which followed the age distribution at the national level. Around two thirds of COs were in the entry-level job group.

**Table 3:** Comparison of deployed COs and participant COs

District	No of deployed COs^a^	No of participant COs	Rate (%)
Rachuonyo South	13	8	62
Gucha	9	6	67
Kenyenya	5	3	60
Nyamache	5	3	60
Nyamira	16	9	56
Nyamira North	5	2	40
North Masaba	13	11	85
Kuria West	12	9	75
Kuria East	7	6	86
Total	85	57^b^	67

^a^Data provided by District Medical Office of Health or district hospital

^b^Corresponds to 16% of COs working at public sector in Nyanza Province (*n* = 356) as of 2009 [16]

**Table 4:** Descriptive statistics of DCE participants

	(*N* = 57)	
	*n*	%
Demographic^a^		
Male^b^	34	60%
Age mean (SD)^c^	33.0	7.6
Currently married	50	88%
Has children	48	84%
Christian	57	100%
Lived in rural area at least 1 year	48	84%
Work experience		
Facility type		
District hospital	26	46%
Sub-district hospital	14	25%
Health centre	13	23%
District Medical Office of Health	4	7%
Employed by government	49	86%
Entry-level job group	36	63%
Years of work experience, mean (SD)	7.3	6.1
Years of work at current facility, mean (SD)	3.0	3.8

^a^Provincial and national level data are rarely available

^b^% of male COs: 61% (national level) [20]

^c^Age distribution: 21–30 years (20%), 31–40 years (50%), 41–50 years (20%) approximately, (national level) [20]

Table 5 presents the regression results for all models. The output from the CL model of the DCE job scenario data is shown in model 1. The coefficients (*β*) indicate the direction and relative importance of the attributes on utility [9]. The coefficient for salary, a continuous valuable, indicates the utility gained per 10% increase above the basic monthly salary for a CO in their first year of service. In model 1, all attributes were statistically significant. COs had high preference for a 1-year guaranteed study leave after 3 years of service (*β* = 2.23, *P* < 0.01) and a 1-year guaranteed study leave after 5 years of service (*β* = 1.64, *P* < 0.01). Further, they preferred good quality health facility infrastructure and equipment to basic health facility infrastructure (*β* = 1.46, *P* < 0.01), followed by superior (*β* = 1.00, *P* < 0.01) and basic (*β* = 0.89, *P* < 0.01) housing allowances and then a 10% salary increase (*β* = 0.39, *P* < 0.01). COs had the least preference for rapid promotion (*β* = 0.34, *P* < 0.05). The model suggests that a 40% (*β* = 1.56) or 50% (*β* = 1.95) salary increase would be required above the CO entry level to be comparable to other attributes.

**Table 5:** DCE regression results

		Model 1^a^	Model 2^a^		Model 3^b^	Model 4^b^
Attribute	Parameter	CL	CL with interaction	Parameter	MXL with salary fixed	MXL with salary random
Salary^c^ (per 10% change above base)	*β*	0.39***	0.40***	Mean	0.44***	0.59***
				SD	–	0.57***
Good quality of facility	*β*	1.46***	1.47***	Mean	1.04***	1.37***
				SD	0.77***	1.07***
Study leave after 5 years	*β*	1.64***	1.80***	Mean	1.42***	1.85***
				SD	0.35	0.66**
Study leave after 3 years	*β*	2.23***	3.15***	Mean	2.11***	2.75***
				SD	0.83***	−1.21***
Basic house allowance	*β*	0.89***	0.90***	Mean	0.80***	1.01***
				SD	0.34	0.65**
Superior house allowance	*β*	1.00***	1.02***	Mean	0.73***	0.95***
				SD	0.00	−0.38*
2 years for promotion	*β*	0.34**	0.40***	Mean	0.23**	0.25*
				SD	−0.04	−0.25
Interaction terms						
Study leave after 5 years × 40 and above			−0.82**			
Study leave after 3 years × 30–39 years old			−1.04***			
Study leave after 3 years × 40 and above			−2.26***			
2 years for promotion × female			0.24*			
Constant		−0.17	−0.19		−0.22*	−0.27*
Model diagnostics						
Number of respondents		57	57		57	57
Number of observations		1 368	1 368		1 368	1 368
Log likelihood		−637.0	−621.0		−324.0	−314.2
AIC		1 290.0	1 266.0		676.1	658.5
BIC		1 331.7	1 328.7		749.2	736.8
Prob > chi^2^		<0.001	<0.001		0.01	<0.001

Although married COs also showed less preference for study leave after 3 years, the result is omitted from the table because there is a correlation between marital status and age group

*CL* conditional logit, *MXL* mixed logit

*P < 0.10; **P < 0.05; ***P < 0.01

^a^Conditional logit model estimated using Stata’s *clogit* command

^b^Mixed logit model estimated using Stata’s *mixlogit* command [31]

^c^Base salary for COs in Kenya at the time of survey administration: KES 19 323 per month (USD 1 = KES 87.31)

CL model 2 includes age group and gender interaction terms which were statistically significant. The sub-group analysis indicates that younger age groups had a significantly higher preference for study leave after 3 years while COs aged 40 and above showed less preference for study leave after 5 years. Female COs had higher preference for rapid promotion. There were no significant differences for other attributes by age or gender.

Next, we turn to the results of the MXL in models 3 and 4. Although the results of both CL and MXL models show that a 1-year guaranteed study leave would have the greatest impact on retention, the MXL models suggest that COs would be more likely to choose a job offering a 30% salary increase (*β* = 1.77) than a job offering good quality health facility infrastructure and equipment (*β* = 1.37). The statistically significant standard deviations in MXL models indicate substantial heterogeneity in COs’ preferences for salary increase, good quality health facility infrastructure and equipment, study leave after 3 years and basic housing allowance.

The results of the WTP calculation for all COs are shown in Table 6. Outputs from CL model 1 were used for WTP calculation because MXL models require larger sample size than available in this study [25] even though the MXL models had better fit than the CL models. The sample sizes for the sub-group analyses were also too small for valid WTP calculations. This analysis explains how much of an entry-level salary (KES 19 323) COs were willing to forgo for another attribute. COs were most willing to sacrifice as much as KES 10 990 in exchange for a 1-year guaranteed study leave after 3 years of service compared with a job posting with no guaranteed study leave. The overall WTP, calculated as a sum of WTPs, can compare retention packages [33]. For instance, if a job offers two incentives: (1) a 1-year guaranteed study leave after 3 years of service and (2) allowance for superior housing (WTP total: KES 15 950), COs are likely to accept the job even if another job offers three incentives: (1) a 30% salary increase, (2) good quality health facility infrastructure and equipment and (3) rapid promotion (WTP total: KES 14 631).

**Table 6:** Willingness to pay estimates (KES)

	Model 1 (CL, all)
	(*N* = 57)
Good quality of facility	7 162 (4 876, 9 447)
Study leave after 5 years	8 064 (5 457, 10 670)
Study leave after 3 years	10 990 (7 699, 14 282)
Basic house allowance	4 372 (2 455, 6 289)
Superior house allowance	4 960 (2 937, 6 983)
2 years for promotion	1 672 (344, 3 000)

Note: Base salary at the time of survey administration: KES 19 323 per month (USD 1 = KES 87.31). Numbers in parentheses are 95% confidence intervals. WTP and confidence intervals were estimated using Hole’s *wtp* command in STATA [46]

## Discussion

Our analysis provides some qualitative insights and quantitative estimates of the likely impact of interventions that aim to improve rural retention among Kenyan COs. The strongest preference was expressed for a 1-year study leave. The Kenyan CO Council has pointed out the inadequate continuous professional development system for COs [34]. The appeal for continuous education has also been observed in the study for MLWs in Tanzania and Malawi [35, 36].

However, considering the resulting increase in workload for remaining staff, a 1-year study leave might be difficult to implement. Further, training interventions can have a negative impact on the supply of workforce. Since the demand for SCOs is not as great as that for RCO [20], further specialist training intervention might lead to the larger imbalance between supply and demand for general COs. Given the report that SCOs feel it is beneath their status to work in out-patient departments when deployed there to cover RCO shortages [21], this type of training intervention should be carefully examined.

A review paper on health worker’s motivation and retention indicates that the improvement of hospital infrastructure and resource availability could increase retention [37]. In our DCE, improved facility quality was the second most important factor influencing COs’ job preferences. This finding is in agreement with results of other DCE studies that show a strong preference for the quality of the facility infrastructure or equipment [26, 37–39]. It also concurs with a motivation study in Kenya and Benin [40].

Salary was an important attribute but only at very high increases above the base level. A 30% increase in salary would have the third highest coefficient. Furthermore, a 30% increase in salary was more preferred to improved facility quality in the MXL model. As salary level went up, the impact on rural retention for COs would increase. This finding concurs with the existing studies that underscore the importance of financial incentives for retention in rural regions [41]. Although the results suggest that COs would be highly responsive to a 30% salary increase, they would value housing allowance more than a 20% salary increase. This finding is consistent with previous studies that suggest that certain levels of salary would be required to improve retention [27, 39].

Housing allowances were also valued by COs. This contrasts with a study conducted in Tanzania, in which CO students did not attribute importance to housing [42]. Moreover, in other DCE studies, practising nurses have less preference for housing attributes than nursing students [27, 43]. Kenyan COs did not value accelerated promotion opportunities as highly as other attributes. This finding concurs with studies carried out with nursing students in Kenya, South Africa and Thailand [27]. Whereas our respondent COs had 7.3 work experience years on average, the majority (63%) remained within the entry-level job group. This suggests that promotion mechanisms do not work effectively, at least in the study sites.

Younger COs showed a strong preference for education opportunities. This finding echoes the DCE conducted for student COs in Tanzania, revealing that COs are eager to gain more knowledge in the early stages of their career [42]. The results also suggest that reactions to incentives vary at the health worker’s career stage [33].

Female COs were more likely to be responsive to promotion than male COs. However, there was no significant difference in other attributes. Existing studies show various consequences. While the DCE study in Tanzania for student COs found females more concerned with facility infrastructure [42], the multi-country DCE study for nurses showed that age and gender are not consistent predictors for choosing a rural post [27]. As there are differences across the studies, it appears important to take local context into account when developing retention strategies.

The sub-group analyses and MXL models suggested substantial heterogeneity among COs for the valuation of most attributes. This would indicate the difficulty of developing effective HRH interventions that apply to all COs. Policymakers could target interventions to different sub-groups of COs or at least consider the differential impact in their planning.

There are several limitations to this study. First, this research was conducted in only some districts in Nyanza Province, and therefore, the findings cannot be generalized to the whole country. Second, the DCE questionnaire in this paper provides only limited description of the attributes, and respondents may interpret these differently. Third, as with all stated preference studies, it is uncertain if respondents will actually select the stated choices. Other factors at various levels may affect the actual job choice decisions [37], and social desirability bias may lead to respondents choosing non-financial attributes rather than financial ones [27]. Lastly, the DCE did not include an opt-out option, which may result in biases in parameter estimates [9].

## Conclusions

Although MLWs including COs are used in many developing countries, more sound evidence is required to gain a better understanding of their motivational factors for retention in rural regions [1]. In a DCE study of 57 Kenyan practising COs, this paper presents data on job preferences. This study confirms that the bundles of intervention, both financial and non-financial, tailored to the local context are more efficient for rural retention than a single intervention [44]. Our study suggests that a study leave intervention would have the strongest impact on COs’ retention in rural Kenya. However, preference for study leave seems to indicate a broader need to consider mechanisms for professional mobility, upgrading skills and status for COs. Further research is needed to examine interventions that can enhance their professional status without leading to a larger imbalance between supply and demand for COs.

## Abbreviations

CO: clinical officer
DCE: discrete choice experiment
GOK: The Government of Kenya
HRH: human resources for health
MLWs: mid-level workers
RCO: registered CO
SCO: specialist CO
USAID: United States Agency for International Development
WHO: World Health Organization
